# Bilateral Adrenal Hyperplasia: Pathogenesis and Treatment

**DOI:** 10.3390/biomedicines9101397

**Published:** 2021-10-05

**Authors:** Benjamin Chevalier, Marie-Christine Vantyghem, Stéphanie Espiard

**Affiliations:** 1 Department of Endocrinology, Diabetology, Metabolism and Nutrition, CHU Lille, F-59000 Lille, France; benjamin.chevalier@chu-lille.fr (B.C.); mc.vantyghem@chru-lille.fr (M.-C.V.); 2 Institut National de la Santé et de la Recherche Médicale (INSERM), U1190, European Genomic Institute for Diabetes (EGID), CHU Lille, F-59000 Lille, France

**Keywords:** bilateral adrenal hyperplasia, primary pigmented micronodular adrenal, primary bilateral macronodular adrenal hyperplasia, Carney complex, Cushing’s syndrome, PKA pathway, *PKRAR1A*, *ARMC5*, paracrine regulation, unilateral adrenalectomy

## Abstract

Bilateral adrenal hyperplasia is a rare cause of Cushing’s syndrome. Micronodular adrenal hyperplasia, including the primary pigmented micronodular adrenal dysplasia (PPNAD) and the isolated micronodular adrenal hyperplasia (iMAD), can be distinguished from the primary bilateral macronodular adrenal hyperplasia (PBMAH) according to the size of the nodules. They both lead to overt or subclinical CS. In the latter case, PPNAD is usually diagnosed after a systematic screening in patients presenting with Carney complex, while for PBMAH, the diagnosis is often incidental on imaging. Identification of causal genes and genetic counseling also help in the diagnoses. This review discusses the last decades’ findings on genetic and molecular causes of bilateral adrenal hyperplasia, including the several mechanisms altering the PKA pathway, the recent discovery of *ARMC5*, and the role of the adrenal paracrine regulation. Finally, the treatment of bilateral adrenal hyperplasia will be discussed, focusing on current data on unilateral adrenalectomy.

## 1. Introduction

Cushing’s syndrome (CS) is characterized by an excess of cortisol production. In 80% of cases, it is due to an over-secretion of the adrenal corticotrophin hormone (ACTH) by a corticotroph pituitary adenoma, or more rarely, by a neuroendocrine tumor. The other 20% is due to a primary overproduction of cortisol by the adrenal glands, with the most frequent etiology being a benign cortisol-secreting adenoma. Other causes of adrenal CS include adrenocortical carcinoma and bilateral adrenal hyperplasia, that account for less than 10% of patients presenting with adrenal CS [1]. Bilateral adrenal hyperplasia may be isolated or part of a syndrome (Table 1). Two groups of bilateral adrenal hyperplasia can be distinguished according to the morphologic presentation: the primary bilateral macronodular adrenal hyperplasia (PBMAH) and the micronodular forms, including the primary pigmented micronodular adrenal (PPNAD) and the isolated micronodular adrenal hyperplasia (iMAD).

**Figure 1:**
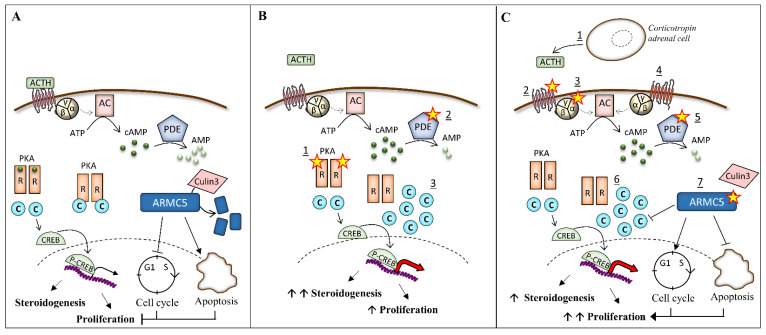
Alteration of protein kinase A (PKA) pathway and ARMC5 in bilateral adrenal hyperplasia. (**A**) In normal adrenocortical cells, ACTH activates the MC2R receptor, leading to the activation of the Gα subunits of the G protein. The latter activates the adenylate cyclase (AC), which converts the ATP in cAMP. The phosphodiesterases (PDE) inactivates cAMP in AMP. The regulatory (R) subunits of the PKA bind the cAMP, leading to the release of the catalytic (C) subunits. The catalytic subunits phosphorylate their targets, including the cAMP Response Element-Binding protein (CREB), which activates genes involved in steroidogenesis. ARMC5 blocks the cell cycle in G1 phase and induces apoptosis. ARMC5 is degraded by Culin3. (**B**) In PPNAD and iMAD, the PKA pathway is activated by (1) mutations in the regulatory subunit R1α of PKA, (2) mutations in phosphodiesterases genes, and (3) duplication of the catalytic subunit Cα have also been described. (**C**) In PBMAH, the PKA pathway is activated by (1) ACTH locally produced by clusters of corticotropin adrenal cells, (2) mutations in the gene coding for MC2R, (3) mutations in gene GNAS coding for Gα, (4) aberrant expression of G-coupled protein receptors, (5) mutations in phosphodiesterase genes, (6) duplication of the catalytic subunit Cα, and (7) ARMC5 mutations, which lead to the activation of the cell cycle and the loss of apoptosis. Moreover, some mutations prevent its binding to Culin3 and its subsequent degradation. In addition, ARMC5 decreases the PKA activity.

Descriptions of familial forms and the bilateral characteristics of the disease suggested that these diseases were genetically determined, which has been confirmed in nearly 70% of the PPNAD and 25% of the PBMAH cases. Most of the genes involved in bilateral adrenal hyperplasia are tumor-suppressor genes. According to Knudson’s theory, one allele is inactivated by a germline mutation (i.e., detectable at the leukocyte level) and the other allele is inactivated at the somatic level (i.e., only present at the tumor level). In addition, most of the genetic or molecular alterations described in these diseases lead to the activation of the protein kinase A (PKA) pathway. The cAMP pathway is a ubiquitous intracellular signaling pathway, regulating several cellular processes, such as proliferation, differentiation, and hormonal activity in endocrine tissues. In adrenals, ACTH binds to its seven-transmembrane domain receptor coupled to the G protein, the MC2R (melanocortin receptor 2), leading to the activation of the Gs subunit. The activated Gs protein stimulates the adenyl cyclase, which synthesizes cAMP. The cAMP binds to the regulatory subunits of the protein kinase A (PKA). The latter dissociates from the catalytic subunits, which then phosphorylate their targets, such as CREB (cAMP Response Binding Protein), a transcription factor involved in adrenal proliferation and steroidogenesis. The cAMP is degraded by phosphodiesterases [2] (Figure 1A). Mutations in the different actors of this pathway or other events, such as the abnormal expression of G-protein coupled receptors are, so far, the most frequent and described alterations in bilateral adrenal diseases (Figure 1B,C). However, in 2013, the discovery of alteration of *ARMC5* (*armadillo repeat containing 5*) in PBMAH brought new insights into the pathogenesis of bilateral adrenal hyperplasia (Figure 1C). 

**Figure 2:**
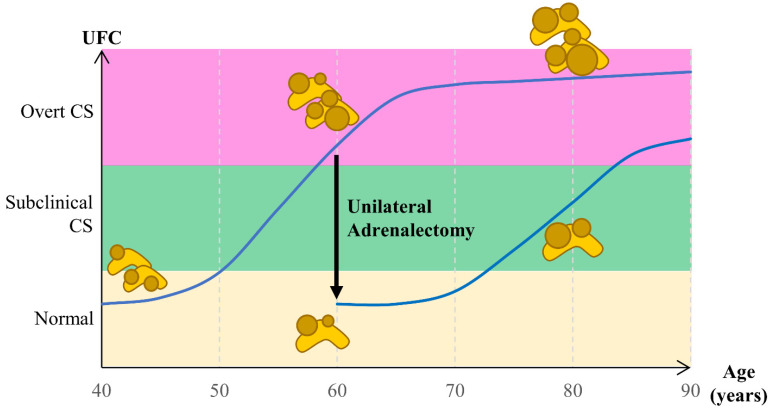
Evolution of cortisol secretion in bilateral adrenal hyperplasia over time and impact of unilateral adrenalectomy.

There is no consensus on the management of bilateral adrenal hyperplasia for several reasons: the disease is rare, patients may present with subclinical CS, and bilateral surgery exposes patients to definitive adrenal insufficiency. Bilateral adrenalectomy used to be the treatment of choice when the decision to treat CS patients was made. Since then, unilateral adrenalectomy has been proposed in both PBMAH and PPNAD, offering interesting results, especially in patients with PBMAH (Figure 2). 

This review states the current data on pathogenesis and treatment of bilateral adrenal hyperplasia. 

## 2. Clinical Features

### 2.1. Micronodular Adrenal Hyperplasia 

#### 2.1.1. PPNAD

Bilateral micronodular adrenal hyperplasia is characterized by micronodules (<1 cm). Histologically, the adrenal cortex shows small pigmented nodules surrounding an atrophic cortex. Adrenal weight and size remain normal [3,4,5]. PPNAD is the most common endocrine manifestation of the Carney complex (CNC). In only 12% of patients, PPNAD appears to be isolated [6]. Contrary to the PPNAD, the isolated micronodular hyperplasia (iMAD) is characterized by a hyperplasia of the internodular tissue [7]. The etiology is unknown in most cases. 

A minority of patients develop PPNAD during the first three years of life, while most will develop the disease during the second and third decades [8]. After puberty, 70% of patients with PPNAD are female [6]. This female predisposition is poorly explained, but the role of estrogen has been suspected. 

The clinical presentation of the disease varies among patients, even in the same family. Patients may present with subclinical or overt CS, with sudden or insidious onset. Cyclic forms with sudden onset of intense hypercortisolism followed by spontaneous remission have also been described [9,10]. Around 25% of the patients with CNC present with overt CS [8]. Lack of suppression of cortisol after a low-dose dexamethasone test is observed in 60% of CNC patients [6]. Interestingly, the autopsies of patients with CNC revealed adrenal dysplasia in all patients, even those who remained asymptomatic [8].

Biologically, fluctuation in the urinary free cortisol (UFC) is frequently observed. A paradoxical elevation of cortisol after four days of a high-dose dexamethasone suppression test has been described in the disease [4], but its sensitivity is low (39%) [11].

A high-resolution computed tomography scan before and after contrast enhancement may detect micronodules that appear as hypodense spots scattered throughout the gland [12]. Occasionally, macronodules are detected, especially in elderly subjects [13]. 

Two cases of adrenocortical cancer [14,15], one case of benign androgen-secreting adenoma [16] and one case of pheochromocytoma [11], have been described in patients with PPNAD. The occurrence of these adrenal tumors is exceptional, and the causality relationship with the PPNAD is unclear. 

#### 2.1.2. Carney Complex

The CNC was first described in 1985 by J. Aidan Carney at the Mayo Clinic [3]. The endocrine and non-endocrine manifestations of CNC are numerous and vary among patients. This disease can be considered as an endocrine and non-endocrine multiple neoplasia syndrome [17]. The prevalence of CNC is difficult to determine because of its rarity. In the most extensive series, including the cohorts of the National Institute of Health (USA), the Mayo Clinic (USA), and the Cochin Hospital (Paris, France), 353 cases were studied [6]. More than 500 patients are currently known to these centers [18], with women representing about 60% of the patients [6,8]. All ethnic groups may be affected [8]. About 70% of cases are familial with autosomal dominant transmission [6,8,19]. The remaining cases are currently considered sporadic. The mean age of diagnosis of the disease is 20 years [8]. In a minority of patients (2%), the diagnosis was made at birth [8]. There is phenotypic variability within families with different ages of expression and onset of manifestations [6,11].

Diagnostic criteria were proposed in 2001 [8]. Manifestations and their frequency are described in Table 2. Cardiac myxomas can affect any cardiac chambers and constitute a significant cause of morbidity and mortality in patients with CNC. Myxomas also develop in breasts and skin. Lentigines and blue naevi are frequent manifestations of the disease but are not very specific in contrast with cutaneous myxomas [10]. Pituitary manifestations include abnormality of the somatotroph and/or lactotroph axis. Most patients have no signs of acromegaly. Pituitary MRI is often normal [20]. Thyroid manifestations usually considered as part of the complex include bilateral macronodules and papillary or follicular carcinomas. The Large Cell Calcifying Sertoli Tumors (LCCST) are the most frequent gonadal lesion described in males. Ovarian cysts are often observed in females [10,11]. Other breast lesions, including adenoma and maybe carcinomas, are observed [11]. Schwannomas in CNC are characterized by the presence of psammoma bodies and melanin. Malignity has been reported in 10% of patients. Other rare tumors include osteochondromyxomas and pancreatic tumors, especially pancreatic intraductal papillary mucinous neoplasm [10].

### 2.2. Macronodular Adrenal Hyperplasia

#### 2.2.1. PBMAH

PBMAH is characterized by the presence of several nodules > 1 cm, usually in both adrenal glands. It was described for the first time in 1964 [21]. The disease affects both sexes, but females may be more represented among sporadic cases. The adrenal hyperplasia is slowly progressing, and the CS appears insidiously after several years of evolution. Thus, diagnosis is often made after the fourth decade. Familial forms have been described.

PBMAH is considered a rare disease, but its prevalence is probably underestimated. Most of the PBMAH have an incidental diagnosis by imaging performed for an unrelated reason. Prevalence of adrenal incidentaloma is estimated at 1% to 5% of abdominal imaging, and 2.7% to 10% of them are bilateral. Subclinical CS is observed in 35% to 40% of these bilateral incidentalomas, and a certain number of cases may correspond to PBMAH [22]. Other patients are diagnosed with PBMAH because of the presence of CS. However, PBMAH is more often responsible for subclinical CS. If published series are biased in including the most severe forms, subclinical CS may concern at least 50% of the cases in the largest published series [23]. Interestingly, the disease is heterogeneous regarding the cortisol over-secretion and the morphologic presentation, even in a family. The question remains if patients with macronodular adrenal hyperplasia on imaging but without biological alteration are indeed PBMAH. 

Differential diagnosis includes bilateral adrenal hyperplasia observed in situations of chronic adrenal overstimulation by ACTH (Cushing’s disease, ectopic secretion of ACTH, congenital adrenal hyperplasia) [24]. Mutation in the *NR3C1* gene, encoding for the glucocorticoid receptor, has also been identified in 5% of a series of patients presenting with bilateral adrenal incidentalomas. These patients do not have features of CS despite an increased urinary free cortisol and an unsuppressed cortisol post-dexamethasone test, consistent with the resistance of cortisol due to the loss of function of the GR. In addition, their ACTH level is unsuppressed. However, they may develop hypertension due to an alteration of the 11β-hydroxysteroid dehydrogenase type 2 activity [25].

In patients diagnosed with PBMAH, there is no specific recommendation for imaging follow-up. At diagnosis, the European Endocrine Society recommends an individual follow-up of each adrenal incidentaloma bigger than 4 cm or spontaneous density above 10 UH (these two features being often observed in PBMAH), with subsequential imaging at 6 months [26]. Regarding the slow progression of the disease, the stability of the hyperplasia will likely be observed. Notably, the occurrence of adrenal carcinoma has not been described so far in patients presenting with PBMAH. It is also not clear if further imaging is needed since the speed of evolution of the disease is unknown. Often, the evolution of hypercortisolism, including its clinical impact and treatment decision, will guide the realization of other CT scans. 

#### 2.2.2. Multiple Tumor Syndromes Associated with Macronodular Adrenal Hyperplasia

MEN1: Multiple endocrine neoplasia type 1 (MEN1) is an autosomal dominant disease linked to mutations in the *MEN1* gene (11q13). It includes primary hyperparathyroidism (95%), pancreatic neuroendocrine tumors (50%), pituitary adenomas (40%), and thymic carcinoid tumors [27]. While adrenal lesions (hyperplasia or nodules) have been reported in up to 50% of patients with MEN1 [28,29,30], the presence of Cushing’s syndrome of adrenal origin remains relatively rare, described in only 0.6% of patients in the French Group of Endocrine tumors cohort [28]. The occurrence of PBMAH has been reported in two patients with MEN1 [28,31]. The causal link between MEN1 and adrenal tumors is supported by the development of adrenal tumors or hyperplasia in mice carrying deletions of specific exons of the *MEN1* gene [32].

Fumarate Hydratase: Autosomal dominant mutations in the *fumarate hydratase (FH)* gene (located on chromosome 1q43) are responsible for hereditary leiomyomatosis-kidney cancer syndrome (HLRCC). FH is an enzyme of the Krebs cycle that allows the conversion of fumarate to malate. Ten HLRCC patients presenting with PBMAH treated by adrenalectomy have been reported [33,34]. One of these PBMAH patients harbored a loss of heterozygosity (LOH) of the gene locus [33], supporting a causal link between the *FH* mutations and the occurrence of PBMAH. Interestingly, a germline *FH* mutation was also characterized in a sporadic case of PBMAH [35].

Familial polyposis coli: Familial polyposis coli or Gardner’s syndrome due to mutation in the *APC* gene is characterized by multiple colonic polyps and colon cancers at an early age. Patients may also present with pigmented retinal lesions, desmoid tumors, osteomas, thyroid nodules or cribriform thyroid cancers, and other malignancies [36]. The development of PBMAH has also been described in these patients [35,37,38]. The observation of second somatic events at the locus of the genes supports a causal link between the *APC* mutations and the occurrence of PBMAH [37,38].

Beckwith–Wiedemann syndrome: Beckwith–Wiedemann syndrome is an imprinting disorder due to genetic or epigenetic alteration of the locus 11p15.5, including *H19*, *IGF2* (Insulin-like growth factor 2), and *CDKN1C* (Cyclin-Dependent Kinase Inhibitor 1C) genes. The phenotype includes overgrowth, neonatal hypoglycemia, congenital malformation, and predisposition to embryonic tumors [39]. If adrenocortical carcinoma is the most frequent adrenal complication of the syndrome, the development of CS due to bilateral nodular enlargement of the adrenal glands has been described in the infant. Histological analysis reveals adrenal cortex cytomegaly, a pathognomonic pattern of this syndrome [40]. 

## 3. Pathogenesis of Bilateral Adrenal Hyperplasia

### 3.1. Alteration of the PKA Pathway 

#### 3.1.1. Alteration of PRKAR1A in PPNAD

Genetic studies have identified two independent loci at chromosomes 17p22–24 and 2p16 in patients with CNC. Currently, no candidate gene has been found at chromosome 2 [41]. On chromosome 17, the R1α regulatory subunit of the protein kinase A (PRKAR1A, cAMP-dependent protein kinase regulatory subunit 1-alpha) was identified in 2000 as the causal gene [42,43]. 

*PRKAR1A* is a tumor-suppressor gene. The transmission of the disease is also autosomal dominant. The mutations are distributed over the 10 coding exons and their adjacent intronic sequences essential for splicing. They are mostly single-base substitutions and short deletions [6,44]. Large deletions of the gene have been more rarely described [45].

In 80% of cases, the mutations cause a premature stop codon and the degradation of the mutant mRNA by nonsense-mediated mRNA decay, leading the haploinsufficiency [6,44,46]. In other cases, the mutant mRNA escapes the degradation process and leads to a shortened, modified, or longer protein according to the type of mutation and its location. Some of these mutant proteins underwent proteasomal degradation [47]. The mutant protein may lose its function and exerts a dominant negative effect on the wild-type protein. Therefore, a second somatic event is not necessary [46]. Of the CNC families, 80%, and 37% of sporadic cases carry a PRKAR1A mutation [6]. Three mutations are found in more than 3 families and can be considered as hotspots: c82C > T (exon 2), c.491_492delTG (exon 5), and c.709(−7–2)del6 (intron 7) [6]. The remaining mutations are mostly unique, identified in only single families. The overall penetrance of the manifestations is about 95% at the age of 50 years. Only two mutations (c.709(−7–2)del6 and c.1A > G) have incomplete penetrance [44].

Some genotype–phenotype correlations have been established [6]:

- Mutations leading to the creation of a mutant protein are associated with a higher number of CNC manifestations.
- Mutations on the exons are more often associated with acromegaly, cardiac myxomas, lentigines, and schwannomas.
- Mutations on the intronic splice sites are associated with a milder phenotype.
- The c.491–492delTG hotspot mutation is more often associated with cardiac myxomas, lentigines, and thyroid tumors.
- The c.709(−7–2)del6 hotspot mutation and the c.1A > G mutation are associated with isolated PPNADs.
- Patients without the PRKAR1A mutation have fewer tumors that appear later in life.

Several mouse models have been developed to study the consequence of PRKAR1A inactivation. The knockout of Prkar1a is lethal at the embryonic stage [48]. Heterozygous mice survive and develop thyroid tumors, schwannomas, and bone lesions [49]. Inactivation of both alleles of the gene after embryonic development leads to follicular adenomas or thyroid hyperplasia, adrenal hyperplasia, lymphomas, hepatocellular carcinomas, and other mesenchymal tumors [50]. Female mice with specific inactivation of Prkar1a in the adrenal cortex develop CS and adrenal hyperplasia and zonation defects [51]. The adrenal hyperplasia is characterized by a zonation defect with regression of the adult cortex and expansion of subcapsular/capsular progenitors that differentiate into steroidogenic cells due to the activation of the PKA pathway. Androgens inhibit PKA signaling and protect males from the development of adrenal dysplasia and CS [52].

Crosstalk between the PKA pathway and other signaling pathways involved in apoptosis and proliferation contributes to tumorigenesis observed with PRKAR1A deficiency [2]. PRKAR1A inactivation leads to the activation of the Wnt/β-catenin pathway and the mTOR pathway, which impairs cell cycle and cell migration [2]. Finally, pigmentation of adrenal nodules is due to the impairment of autophagy, leading to the accumulation of lipofuscin at the nodules [53].

#### 3.1.2. Alteration of Other Genes Involved in the PKA Pathway

PRKACA: In a cohort of 35 patients with bilateral adrenal hyperplasia, germline amplification (duplication or triplication) of PRKACA has been described in 5 patients, including 3 patients with PPNAD and 2 patients with PBMAH, a mother and her son [54,55]. This amplification leads to the overexpression of the catalytic subunits, resulting in an increased PKA activity [54]. This locus’ duplication has also been reported in three patients presenting with PBMAH, including one adult, a mother, and her son [54,55]. Interestingly, a 2-year-old child with PPNAD had a history of neonatal hypoglycemia and macroglossia [55].

MC2R: Mutations in the ACTH receptor (MC2R) have been described in two sporadic cases of PBMAH [56,57]. No other study has identified mutations in this gene, which makes it difficult to conclude that there is a causal link between these mutations and the occurrence of the disease.

Phosphodiesterases: The presence of variants of the PDE11A (Phosphodiesterase 11A) gene appears to confer a genetic predisposition to PBMAH. In a series of 46 patients, the frequency of PDE11A variants was higher than in the control population [58]. This observation was later confirmed in an independent cohort [59]. In vitro analysis of the two most frequent variants showed an increase in cAMP levels and PKA activity [59]. 

In few children with iMAD, mutations in the PDE11A [60] and the PDE8B (Phosphodiesterase 8A) genes [61] are responsible for the disease. In addition, in patients with CNC, PDE11A variants may confer a predisposition to developing LCCST and PPNAD, particularly in men [62].

GNAS: In children, the most common cause of Cushing’s syndrome of adrenal origin is the McCune Albright syndrome (MAS), related to post-zygotic mutations in the GNAS gene, encoding the Gαs subunit of the heterotrimeric G protein [63]. The disease is characterized by fibrous dysplasia of the bones, the presence of café au lait spots, and endocrine activation, the most common leading to precocious puberty. CS due to bilateral adrenal hyperplasia may occur within the first year of life, revealing the syndrome. Histological analysis reveals a bi-morphic pattern with diffuse and nodular hyperplasia, cortical atrophy, and the persistence of the fetal cortex [64]. In these situations, CS is often the first manifestation of the syndrome. It typically occurs within the first year of life and may resolve spontaneously. Interestingly, somatic mutations of GNAS have also been described in the nodules of PBMAH [65].

#### 3.1.3. Aberrant Expression of G-Coupled Protein Receptor in PBMAH

Abnormal cortisol secretion due to the activation of G-coupled protein receptors other than MC2R was one of the first pathogenic mechanisms demonstrated in PBMAH. In 1992, a food-dependent CS [66,67] due to an abnormal expression of the gastric inhibitory polypeptide (GIP) receptor was described. Interestingly, patients with GIP response usually have a hypo-cortisolism in fasting, especially at 8 am, contrasting with the CS [66,67]. Since then, several publications have reported an abnormal cortisol response to various stimuli, suggesting an abnormal expression of different receptors [68], including:

- Eutopic receptors (normally expressed in adrenocortical cells), such as the vasopressin V1 receptor, the luteinizing hormone/human chorionic gonadotropin (LH/HCG) receptor, the serotonin 5-HT4 receptor, and the leptin receptor.
- Ectopic receptors (absent in normal adrenocortical cells), such as the GIP receptor, the vasopressin V2 and V3 receptors, the serotonin 5-HT7 receptor, the glucagon receptor, the beta-adrenergic receptor, and the angiotensin II AT1-receptor.

The presence of these receptors can be clinically assessed by a combination of biological tests [69] (Table 3). In a series of 32 patients, 87% of them presented with at least one abnormal response. The most frequent response was to posture (67%), metoclopramide (56%), and glucagon (47%). Food-response concerned only 12% of patients [70]. Besides the GIP and the LH/HCG receptors’ abnormal expression, which has been shown to induce CS during pregnancy or after menopause, the presence of these receptors does not affect the presentation of the disease [71]. In a patient presenting with bilateral adrenal incidentaloma, an abnormal response may argue for the diagnosis of PBMAH, but such abnormal responses can also be observed in other adrenal tumors [68,72].

Abnormal expression or overexpression of these receptors has been confirmed by quantitative PCR [68] or transcriptomic analysis [73,74]. In most cases, the abnormal expression leads to the activation of the PKA pathway. In primary adrenocortical cells from patients presenting with an abnormal cortisol response, an increase of cortisol production after stimulation by the corresponding ligand is observed [66,68,75,76,77,78]. In addition, transplantations of bovine adrenocortical cells expressing the GIP or the LH/HCG receptors beneath the kidney capsule of adrenalectomized immunodeficient mice led to hyperplasia of the graft hyperplasia and CS [79,80].

The mechanism leading to this aberrant expression, which is likely to be an early event in PBMAH [74,81], is unknown for most receptors [68]. At the germline level, no genetic alteration of these receptors has been described. An Armc5+/− mice study suggests that *ARMC5* inactivation may be responsible for the abnormal expression of the alpha-2 adrenergic receptor and the AVP-R1A receptor [82]. At the somatic level, duplication of the locus, including the GIP receptor, has been shown in food-dependent cortisol-secreting adenomas and one patient with food-dependent PBMAH. In two adenomas, the duplicated region was rearranged with other chromosome regions including glucocorticoids response elements, thus driving the abnormal expression of the translocated GIPR [83]. In transcriptomic analysis, food-dependent Cushing PBMAH cluster together, suggesting common molecular alterations [84].

### 3.2. Mutation of ARMC5 in PBMAH 

#### 3.2.1. Genetic Mutations of *ARMC5*

In 2013, mutations in the *ARMC5* gene (*Armadillo repeat containing 5*) had been identified by an integrated genomics approach as responsible for PBMAH [85]. LOH at the short arm of chromosome 16 was first identified by a single-nucleotide polymorphism (SNP) array as a frequent event in adrenal tumor tissues. Whole-genome sequencing and Sanger sequencing of paired leukocytes–somatic DNA subsequently allowed the identification of *ARMC5*, located at the chromosome 16p, as responsible for PBMAH in a series of 33 patients [85]. Several series of sporadic cases from different continents have since confirmed that *ARMC5* mutations account for 25% of the PBMAH, except in Japan, where the prevalence may be higher [23,86,87,88,89]. 

*ARMC5* mutation leads to more severe disease with higher hypercortisolism, larger adrenal hyperplasia, and a higher number of nodules [23]. Patients present more frequently with hypertension [23], most likely because of the more severe hypercortisolism, but *ARMC5* variants have also been associated in African Americans with low renin hypertension, higher fasting glucose, and HbA1c [90,91]. In addition, co-secretion of cortisol and aldosterone has been reported in one patient [90]. Due to the severity of the disease, patients carrying *ARMC5* mutations undergo surgery more often [23], explaining the higher prevalence of *ARMC5* mutations in series including only operated patients. Interestingly, no food response has been observed in *ARMC5*-mutated patients, while a response to vasopressin or orthostatism can be observed [23,92,93].

*ARMC5* mutations are responsible for nearly 80% of the familial forms [88,89,92,93,94]. Familial studies suggest that the penetrance of the disease is high but not complete [88,92]. In addition, the phenotype is variable and limited in some relatives to moderate adrenal CT scan alteration or subtle alteration of the pituitary-adrenal axis, even at an advanced age [88,94]. 

Meningiomas in *ARMC5*-mutated patients have been described [88,93,94,95,96]. The observation in the meningeal tumor of a LOH of the locus or a mutation on the second allele supports that *ARMC5* mutations are responsible for meningioma [94,95].

*ARMC5* inactivation followed the two-hits models observed for tumor-suppressor genes. The described mutations are distributed along the coding sequences. A multitude of mutations have been described since 2013, suggesting that most of them are unique for one patient and his family. There are no true hotspots, even though some mutations have been found by several teams [97]. Deletions of the gene have been more rarely reported [23,98].

#### 3.2.2. Function of ARMC5

The function of *ARMC5* was unknown when it was characterized as a causal gene of PBMAH in 2013. The ARMC5 protein is part of the Armadillo repeat containing gene family. Its structure contains two highly conserved domains involved in protein–protein interaction: the armadillo repeat domain and a broad complex Tramtrack bric-a-brac/Pox virus and zinc finger (BTB/POZ) domain. The protein is ubiquitously expressed [99]. The first functional studies of the ARMC5 mutant protein suggested that ARMC5 is involved in apoptosis. ARMC5 mutant overexpression in human adrenocortical cell lines leads to the loss of the apoptosis normally observed with the wild-type protein [23,85,100]. 

Inactivation of *ARMC5* in vitro decreases the expression of genes involved in steroidogenesis and cortisol production [85,100]. Interestingly, transcriptome analysis has previously shown a reduced expression of steroidogenic enzymes [101], while a decrease of cortisol production has been demonstrated in primary cultures of PBMAH cells [73]. Therefore, it is suggested that the CS will appear when the adrenal mass will be big enough to balance the decreased steroidogenesis observed at the cellular scale [97]. Recent data consistently suggest that adrenal gland size correlates with 17-hydroxycorticosteroids in patients carrying pathogenic variants of *ARMC5* [102]. 

Knockout of Armc5 in mice has a high lethality rate at the embryonic stage [82,103]. Armc5 heterozygote mice (Armc5+/−) develop hypocorticosteronemia at 12 months of age, supporting in vitro data showing that *ARMC5* deficiency decreases steroidogenesis. Interestingly, a decrease in the expression of Prkaca was observed in these mice [99]. Similarly, a decreased expression of PRKACA and a decreased PKA activity have been previously described in the largest nodules of PBMAH [104]. However, this hypocorticosteronemia is transient in the Armc5+/− mice, and one third of the mice finally develop hypercorticosteronemia at 18 months of age. Armc5+/− mice do not develop macronodules but do develop features of cortex damage [99], while adrenal hyperplasia has been observed in Armc5−/− mice [103]. 

*ARMC5* is also involved in cell cycle regulation. ARMC5 interacts with Cullin 3 via its BTB/POZ domain, leading to the proteasomal degradation of ARMC5. Interestingly, *ARMC5* overexpression alters the G1-S progression, and Cullin 3 blocks this effect. Mutations in the BTB domain of ARMC5 affect its degradation and its action on the cell cycle [105]. Finally, the involvement of *ARMC5* in T-cell function has also been suggested by another knockout mice model study [103].

### 3.3. Paracrine and Autocrine Factors in PBMAH

Paracrine and autocrine regulation of adrenal glands by peptides or neurotransmitters secreted by chromatin cells, nerve endings, or immune cells has been previously demonstrated [106,107,108]. 

Chromaffin cells in the medulla produce ACTH locally [109]. In PBMAH, some specific clusters of cortical cells are also able to produce ACTH. These cells express the proopiomelanocortin and the proconvertase 1. Their immunohistochemical profile suggests that they are steroidogenic cells with a gonadal differentiation [110]. Veinous adrenal catheterism in two patients presenting with PBMAH demonstrated the ACTH production by the adrenals. Interestingly, the ACTH immunostaining was correlated with the cortisol level in a series of patients [110]. Thus, the appearance of this cluster of cells is likely to contribute to the onset of CS in patients. In addition, this observation explains why the ACTH level is often not suppressed in patients with PBMAH despite hypercortisolism. After this publication, the term ACTH-independent macronodular adrenal hyperplasia previously used has been replaced by PBMAH [111]. The exact origin of these cells and the role of *ARMC5* in their appearance must be elucidated. 

In addition to ACTH, local production of serotonin by the perivascular mastocyte cells located in the subcapsular area of the adrenal cortex is observed. This local serotonin stimulates the production of aldosterone [108,112]. In PBMAH, the characterization of clusters of cells producing serotonin in the center of the nodules, together with the demonstration of aberrant expression of 5-HT4 and 5-HT7 receptors, suggest an abnormal 5-HT paracrine pathway contributing to cortisol hypersecretion [75,108]. The presence of cells expressing the chromogranin A [110] or AVP [75] in PBMAH tissues also suggests a local stimulation of illegitimate β or α2-adrenergic and AVP receptors [108].

## 4. Treatment

### 4.1. Decision for Treatment in Bilateral Adrenal Hyperplasia

In patients with overt CS, the indication to treat to normalize the cortisol level is consensual [113]. However, in patients with bilateral adrenal hyperplasia, bilateral adrenalectomy induces definitive adrenal insufficiency that requires lifelong hormonal replacement and, therefore, exposes patients to the life-threatening adrenal crisis. 

Definitive adrenal insufficiency also exposes patients to increased morbidity and mortality due to cardiovascular diseases and infections [114,115,116]. Since the CS is often subclinical in bilateral adrenal hyperplasia, the benefit/risk balance is also controversial. In addition, the benefit of surgical treatment of adrenal incidentaloma with subclinical CS compared to the medical control of the cardiovascular risk factors has not been clearly demonstrated yet [117]. A meta-analysis including 26 studies showed that patients with subclinical CS undergoing adrenalectomy improved their cardiovascular risk factors, especially hypertension and diabetes compared to conservative management [118]. However, most of these previous studies are retrospective and used different definitions of subclinical CS. Therefore, prospective studies are still needed to confirm the benefits of surgical treatment of subclinical CS. 

Finally, it is unclear if the results of studies on adrenal incidentaloma apply to patients with bilateral adrenal hyperplasia. Particularly, the onset of PPNAD is younger than the age of diagnosis of adrenal incidentaloma, and the balance between the long-term consequences of definitive adrenal insufficiency versus long-term consequences of subclinical CS must be determined. 

Therefore, unilateral adrenalectomy appears as an interesting option to treat patients with bilateral adrenal hyperplasia (Figure 2). 

### 4.2. Surgical Treatment 

#### 4.2.1. Surgical Treatment of PPNAD

Bilateral adrenalectomy used to be considered as the treatment of choice for patients with overt CS and adrenal hyperplasia [113]. In patients with PPNAD, bilateral adrenalectomy is usually preferred in adult patients. It leads to the complete remission of the CS in almost every case. Incomplete resection with residual tissue within the operative fields is the cause of the persistence of the disease [119]. 

Nevertheless, unilateral adrenalectomy has been proposed in patients with PPNAD to avoid definitive adrenal insufficiency. In a recent review of all published cases or series, the initial success rate was evaluated at 66% (32 of the 48 reported patients) [120]. Most of the patients had overt CS. The selection of the side of the adrenalectomy was based on the eventual presence of a macronodule or an asymmetry of the uptake on ^131^I- norcholesterol scintigraphy. Only 7 of 32 patients considered in initial remission needed contralateral adrenalectomy because of recurrence of the CS. Among the 25 patients that did not need completion of the surgery, follow-up was unavailable for 9 patients [121,122,123], and 1 patient refused the surgery [124]. One patient presented with adrenocortical carcinoma, and PPNAD was discovered on anatomopathological examination. She would later die from her carcinoma 6 months after surgery [14]. Follow-up was limited to less than one year in two cases [125,126]. Finally, only 12 patients (48% of the patients in initial remission) did not present recurrence of CS after a follow-up between 16 to 113 months [127,128]. 

Taken together, these results hence suggest that unilateral adrenalectomy could be an interesting option in selected patients presenting with PPNAD, especially in younger patients. However, interpretations of these data should be made with caution. In addition to the lack of biological and clinical data in several reports, one major limitation is the different criteria used to define remission. In some cases, alteration of the adrenal-pituitary axis tests, especially an abnormal circadian rhythm, was still observed [120]. Interestingly, only a few patients seem to present with corticotroph deficiency after unilateral adrenalectomy [129]. Finally, the success rate of unilateral adrenalectomy may be overestimated by the preferential publication of successful case reports. In the largest series, including 17 patients, only 35% of patients presented with initial remission. 

Urinary free cortisol (UFC) level increases progressively with the evolution of adrenal hyperplasia. Unilateral adrenalectomy leads to a reduction of the adrenal mass and a reduction of the UFC at levels equivalent to those 10 to 20 years earlier.

#### 4.2.2. Surgical Treatment of PBMAH

Regarding the late onset of the disease and the higher frequency of subclinical CS compared to PPNAD, unilateral adrenalectomy instead of bilateral adrenalectomy appears particularly interesting in patients with PBMAH. Unilateral adrenalectomy has also been proposed in PBMAH patients since the late 1990s. Currently, 23 case reports and small series totaling 117 patients have been reported [120]. Initial remission was observed in 93% of the patients. Remission of the hypercortisolism varies from several months to up to 15 years for the most extended follow-up available. Only 15% of the reported patients presented with recurrence. Contralateral adrenalectomy was performed in most of them within a median time of 72 months [120]. These retrospective studies are exposed to the same bias as those discussed in the literature on PPNAD, but, undeniably, unilateral adrenalectomy seems to be efficient in most patients presenting with PBMAH.

UFC normalizes in almost every patient. A division of the UFC by nearly 8 has been described in patients one month after the unilateral adrenalectomy. In contrast, unilateral adrenalectomy leads to the division by a bit more than 2 of the adrenals. This observation may be explained in patients with high cortisol levels by the saturation of the corticosteroid-binding globulin that leads to the rapid increase of the ratio of free/bound cortisol. The division by 2 of the cortisol production will also lead to a bigger division of the UFC level [130]. Adrenal insufficiency due to corticotroph deficiency is observed in about one-third of patients. This deficiency may persist for several years in some patients [120]. Interestingly, after unilateral adrenalectomy, the Synacthen test may provide a false positive response due to the remaining hyperplastic tissue being overactivated by ACTH via MC2R [130]. 

Removal of the largest adrenal is usually performed. Nordiodocholesterol scintigraphy shows in all cases a maximum uptake in the largest glands [130]. Few studies have evaluated the interest in adrenal venous sampling [131,132]. However, this test is invasive and requires a specialized center with a trained radiologist. 

In most studies, improvement of cortisol-related comorbidities, including obesity, diabetes, and hypertension, is reported in patients treated by unilateral adrenalectomy [130,133]. In contrast, a recent retrospective study suggested that unilateral adrenalectomy may lead to insufficient biochemical remission compared to bilateral adrenalectomy [133]. Indeed, post-dexamethasone cortisol or midnight salivary cortisol may remain increased in some patients. 

More recently, adrenal-sparing surgery (removal of one adrenal and up to two-thirds of the other one) as performed in patients with some genetic predisposition to pheochromocytoma has been proposed [134]. The interest of this surgery compared to unilateral adrenalectomy needs to be evaluated.

### 4.3. Medical Treatment 

All patients presenting with adrenal hyperplasia and subclinical CS must have a regular screening, symptomatic treatment of comorbidities, and strict control of cardiovascular risk factors.

The presence of illegitimate receptors may offer the possibility to propose a specific medical therapy. Beta-blockers, especially propranolol, have been proposed in case of cortisol response to posture. Long-term control of hypercortisolism with such therapy has been described, but the side effects of the treatment are limiting factors [135,136,137]. Somatostatin analog is initially efficient in some cases of food-dependent CS, but escape is most often observed [135,138,139]. A treatment by leuprorelin, a GnRH agonist, has shown a long-term response in the case of PBMAH presenting with a LH/HCG response [139]. The presence of several different responses in one patient is one factor that limits the total response to the monotherapy.

Anti-cortisolic treatment can be temporarily used for patients presenting with overt CS waiting for surgery. Long-term treatment by steroidogenesis inhibitors, including ketoconazole, metyrapone, or mitotane, has been proposed in both PBMAH [140,141] and PPNAD [142] patients presenting with overt CS. Treatment with metyrapone administered at the end of the afternoon and at bedtime has been proposed in patients with adrenal incidentalomas and subclinical CS to try to restore the normal circadian rhythm [143]. The osilodrostat, a new powerful 11β-hydroxylase inhibitor, may be an interesting alternative for long-term treatment of PBMAH and PPNAD. Studies are needed to confirm the long-term efficacity and the good tolerance in these indications.

## 5. Conclusions

Significant advances in the understanding of bilateral adrenal hyperplasia pathogenesis have been made in the last three decades. The PKA pathway so far appears to be the main pathway dysregulated in bilateral adrenal hyperplasia, especially PPNAD. In agreement with its effect on steroidogenesis and cellular differentiation, its alterations lead to CS, and the slowly growing hyperplasia and nodularity. In the last decade, the discovery of *ARMC5* mutations brought new insight into the pathogenesis of PBMAH. The role of *ARMC5* may take another decade to be fully understood since its role appears to be complex, involving cell cycle, proteasomal degradation, and crosstalk with other pathways, such as the PKA pathway. In the coming years, whole-genome sequencing will likely bring new candidates to explain the nearly 30% and 75% unresolved cases of micronodular adrenal hyperplasia and PBMAH, respectively. Progress in understanding other mechanisms such as paracrine regulation in adrenal glands will also help to understand the evolution of this group of diseases.

In all patients presenting bilateral adrenal hyperplasia, genetic screening should be offered, including at least the analysis of *PRK1R1A* in patients with PPNAD and the analysis of *ARMC5* in patients with PBMAH. Complementary analysis of phosphodiesterases genes and *PRKACA* should be discussed. Next-generation sequencing allows screening these genes in a unique array, including the research of amplification of *PRKACA*. Genetic counseling must be performed in first-degree relatives of *PRKAR1A* mutation carriers and should be discussed in first-degree relatives of *ARMC5* mutation carriers.

Bilateral adrenalectomy is an effective treatment, but clinicians must weigh the arising complications of adrenal insufficiency against the complications of hypercortisolism, especially in patients with subclinical CS. Unilateral adrenalectomy appears to be a compelling alternative in patients presenting with bilateral adrenal hyperplasia, particularly those with PBMAH. Prospective studies are needed to compare medical therapy and adrenalectomy in patients presenting subclinical CS. Both short- and long-term evaluations of the complications of hypercortisolism need to be carried out in these studies.

## Figures and Tables

**Table 1:** Germline defect associated with adrenal hyperplasia. ^1^ NA: Not Applicable: the described mutations may lead only to adrenal hyperplasia, but they have been described only in case reports.

Gene	Genetic	Function	Phenotype	Frequency of the Adrenal Hyperplasia in Case of Mutations of the Gene
*PRKAR1A*	Unique inactivating mutations spread along the gene. 3 hotspots (c.709(−7–2)del6, c.491–492delTG, c82C > T). Large deletions described	Regulatory subunit R1α of the PKA. Inhibition of PKA pathway	Isolated PPNAD (~12%) Carney complex: cardiac, skin and breast myxomas, lentigines, pituitary adenoma or hyperplasia (GH +/− PRL), LCCST, osteochondromyxoma, schwannomas	26% to 60% [1,2,3]
*PRKACA*	Amplification of the gene	Catalytic subunit Cα of the PKA. Activation of PKA pathway	PBMAH Macroglossia?	NA ^1^
*GNAS*	Post-zygotic activating mutations Two hotspots (p.R201H and p.C174Y)	G protein subunit alpha stimulating. Activation of PKA pathway	Macronodular adrenal hyperplasia Mc Cune Albright syndrome: precocious puberty, Café-au-lait spot, polyostotic fibrous dysplasia, somatotroph adenoma or prolactinoma, multinodular goiter, hyperthyroidism	Near 5% [4,5]
*PED8B* *PDE11A*	Unique inactivating mutations	Phosphodiesterase type 8B and 11A. Inactivation of PKA pathway	iMAD	NA ^1^
*MC2R*	Unique activating mutations	ACTH receptor. Activation of the PKA pathway.	PBMAH	NA ^1^
*ARMC5*	Unique inactivating mutations spread along the gene.	Potentially control apoptosis and cell cycle. Interaction with PKA pathway and steroidogenesis?	PBMAH Meningioma (several cases described)	ND, High penetrance described in families
*MEN1*	Unique inactivating mutations spread along the gene. Large deletions	Scaffold protein controlling gene transcription and many other cellular functions, such as proliferation	PBMAH Pituitary adenoma Primary hyperparathyroidism Neuroendocrine tumors	Case reports
*FH*	Unique inactivating mutations spread along the gene.	Krebs cycle	HLRCC: leiomyomatosis, renal cell cancer	Estimated at 0.8% [6]
*APC*	Unique inactivating mutations spread along the gene.	Inhibition of Wnt/β-catenin pathway	PBMAH Familial adenomatous polyposis	Case reports

**Table 2:** Manifestations of Carney Complex.

Clinical Features	Frequency (%) [1,2,3]	Age at Diagnosis (Years) [2,3]
PPNAD	45–68	Median: 25 Bimodal age distribution: in the first 3 years of life or in the 2nd and 3rd decades
Skin lesion		
Lentigines	56–70	From birth or appear progressively, fade after the 4th decade
Blue naevi	17–50	May appear in early childhood years
Cutaneous myxoma	20–45	May appear within the first 10 years of life
Cardiac myxoma	23–53	Median: 29 Described in the first years of life
Hypersomatotropism	10–19	Median: 35
Thyroid tumors	5–25	May appear within the first 10 years of life
Psammomatous melanotic schwannoma	8–10	Median: 35
Osteochondromyxoma	2–6	Described in the first years of life but also in adults
Breast lesions	19–42	Breast myxomas may appear in childhood
LCCSCT	33–41	Median: 28 Described from the first years of life

**Table 3:** Aberrant expression of G-coupled protein receptor in PBMAH, and their screening protocols. Adapted from [69,70,71]. After stimulation, a change in plasma cortisol > 25% from baseline was defined as a response (between 25% and 49%: partial response, >50% or greater: positive response).

Receptor	Ligand	Diagnostic Tests
***Ectopic receptors***		
GIP receptor	GIP	Standard mixed meal, IV GIP infusion
V2R/V3 receptor	AVP/Anti-diuretic hormone	Supine-to-upright posture test, AVP/IM/SC desmopressin infusion (terlipressin)
β-adrenergic receptor	β-epinephrine	Insulin hypoglycemia IV isoproterenol infusion
AT1 receptor	Angiotensin 2	Supine-to-upright posture test, IV angiotensin 2 infusion
5-HT7 receptor	Serotonin	Metoclopramide administration
Glucagon receptor	Glucagon	IV glucagon infusion
***Eutopic receptors***		
V1R receptor	AVP/Anti-diuretic hormone	Supine-to-upright posture test IM desmopressin infusion (terlipressin)
5-HT4 receptor	Serotonin	Metoclopramide administration
LH/HCG receptor	LH/HCG	IV GnRH infusion IM LH or HCG infusion
PRL receptor	Prolactin	Chlorpromazine administration IV TRH infusion

AVP: Arginine Vasopressin, AT1 receptor: Angiotensin 2 Type 1 receptor, GnRH: Gonadotropin-Releasing Hormone, PRL: Prolactin, TRH: Thyrotropin-Releasing Hormone.

## References

[1] Lodish M., Stratakis C.A. (2016). A Genetic and Molecular Update on Adrenocortical Causes of Cushing Syndrome. Nat. Rev. Endocrinol..

[2] Yu B., Ragazzon B., Rizk-Rabin M., Bertherat J. (2012). Protein Kinase A Alterations in Endocrine Tumors. Horm. Metab. Res. Horm. Stoffwechselforschung Horm. Métabolisme.

[3] Carney J.A., Gordon H., Carpenter P.C., Shenoy B.V., Go V.L. (1985). The Complex of Myxomas, Spotty Pigmentation, and Endocrine Overactivity. Medicine (Baltimore).

[4] Stratakis C.A., Sarlis N., Kirschner L.S., Carney J.A., Doppman J.L., Nieman L.K., Chrousos G.P., Papanicolaou D.A. (1999). Paradoxical Response to Dexamethasone in the Diagnosis of Primary Pigmented Nodular Adrenocortical Disease. Ann. Intern. Med..

[5] Shenoy B.V., Carpenter P.C., Carney J.A. (1984). Bilateral Primary Pigmented Nodular Adrenocortical Disease. Rare Cause of the Cushing Syndrome. Am. J. Surg. Pathol..

[6] Bertherat J., Horvath A., Groussin L., Grabar S., Boikos S., Cazabat L., Libe R., René-Corail F., Stergiopoulos S., Bourdeau I. (2009). Mutations in Regulatory Subunit Type 1A of Cyclic Adenosine 5′-Monophosphate-Dependent Protein Kinase (PRKAR1A): Phenotype Analysis in 353 Patients and 80 Different Genotypes. J. Clin. Endocrinol. Metab..

[7] Stratakis C.A., Boikos S.A. (2007). Genetics of Adrenal Tumors Associated with Cushing’s Syndrome: A New Classification for Bilateral Adrenocortical Hyperplasias. Nat. Clin. Pract. Endocrinol. Metab..

[8] Stratakis C.A., Kirschner L.S., Carney J.A. (2001). Clinical and Molecular Features of the Carney Complex: Diagnostic Criteria and Recommendations for Patient Evaluation. J. Clin. Endocrinol. Metab..

[9] Gunther D.F., Bourdeau I., Matyakhina L., Cassarino D., Kleiner D.E., Griffin K., Courkoutsakis N., Abu-Asab M., Tsokos M., Keil M. (2004). Cyclical Cushing Syndrome Presenting in Infancy: An Early Form of Primary Pigmented Nodular Adrenocortical Disease, or a New Entity?. J. Clin. Endocrinol. Metab..

[10] Espiard S., Bertherat J. (2013). Carney Complex. Front. Horm. Res..

[11] Espiard S., Vantyghem M.-C., Assié G., Cardot-Bauters C., Raverot G., Brucker-Davis F., Archambeaud-Mouveroux F., Lefebvre H., Nunes M.-L., Tabarin A. (2020). Frequency and Incidence of Carney Complex Manifestations: A Prospective Multicenter Study With a Three-Year Follow-Up. J. Clin. Endocrinol. Metab..

[12] Courcoutsakis N.A., Tatsi C., Patronas N.J., Lee C.-C.R., Prassopoulos P.K., Stratakis C.A. (2013). The Complex of Myxomas, Spotty Skin Pigmentation and Endocrine Overactivity (Carney Complex): Imaging Findings with Clinical and Pathological Correlation. Insights Imaging.

[13] Groussin L., Jullian E., Perlemoine K., Louvel A., Leheup B., Luton J.P., Bertagna X., Bertherat J. (2002). Mutations of the PRKAR1A Gene in Cushing’s Syndrome Due to Sporadic Primary Pigmented Nodular Adrenocortical Disease. J. Clin. Endocrinol. Metab..

[14] Anselmo J., Medeiros S., Carneiro V., Greene E., Levy I., Nesterova M., Lyssikatos C., Horvath A., Carney J.A., Stratakis C.A. (2012). A Large Family with Carney Complex Caused by the S147G PRKAR1A Mutation Shows a Unique Spectrum of Disease Including Adrenocortical Cancer. J. Clin. Endocrinol. Metab..

[15] Morin E., Mete O., Wasserman J.D., Joshua A.M., Asa S.L., Ezzat S. (2012). Carney Complex with Adrenal Cortical Carcinoma. J. Clin. Endocrinol. Metab..

[16] Hofland J., de Herder W.W., Derks L., Hofland L.J., van Koetsveld P.M., de Krijger R.R., van Nederveen F.H., Horvath A., Stratakis C.A., de Jong F.H. (2013). Regulation of Steroidogenesis in a Primary Pigmented Nodular Adrenocortical Disease-Associated Adenoma Leading to Virilization and Subclinical Cushing’s Syndrome. Eur. J. Endocrinol. Eur. Fed. Endocr. Soc..

[17] Stratakis C.A., Kirschner L.S., Carney J.A. (1998). Carney Complex: Diagnosis and Management of the Complex of Spotty Skin Pigmentation, Myxomas, Endocrine Overactivity, and Schwannomas. Am. J. Med. Genet..

[18] Rothenbuhler A., Stratakis C.A. (2010). Clinical and Molecular Genetics of Carney Complex. Best Pract. Res. Clin. Endocrinol. Metab..

[19] Carney J.A., Hruska L.S., Beauchamp G.D., Gordon H. (1986). Dominant Inheritance of the Complex of Myxomas, Spotty Pigmentation, and Endocrine Overactivity. Mayo Clin. Proc. Mayo Clin..

[20] Kirschner L.S. (2010). PRKAR1A and the Evolution of Pituitary Tumors. Mol. Cell. Endocrinol..

[21] Kirschner M.A., Powell R.D., Lipsett M.B. (1964). Cushing’s syndrome: Nodular cortical hyperplasia of adrenal glands with clinical and pathological features suggesting adrenocortical tumor. J. Clin. Endocrinol. Metab..

[22] Espiard S., Benomar K., Loyer C., Vahé C., Vantyghem M.-C. (2018). European Recommendations for the Management of Adrenal Incidentalomas: A Debate on Patients Follow-Up. Ann. Endocrinol..

[23] Espiard S., Drougat L., Libé R., Assié G., Perlemoine K., Guignat L., Barrande G., Brucker-Davis F., Doullay F., Lopez S. (2015). ARMC5 Mutations in a Large Cohort of Primary Macronodular Adrenal Hyperplasia: Clinical and Functional Consequences. J. Clin. Endocrinol. Metab..

[24] Sohaib S.A., Hanson J.A., Newell-Price J.D., Trainer P.J., Monson J.P., Grossman A.B., Besser G.M., Reznek R.H. (1999). CT Appearance of the Adrenal Glands in Adrenocorticotrophic Hormone-Dependent Cushing’s Syndrome. AJR Am. J. Roentgenol..

[25] Vitellius G., Trabado S., Hoeffel C., Bouligand J., Bennet A., Castinetti F., Decoudier B., Guiochon-Mantel A., Lombes M., Delemer B. (2018). Significant Prevalence of NR3C1 Mutations in Incidentally Discovered Bilateral Adrenal Hyperplasia: Results of the French MUTA-GR Study. Eur. J. Endocrinol..

[26] Fassnacht M., Arlt W., Bancos I., Dralle H., Newell-Price J., Sahdev A., Tabarin A., Terzolo M., Tsagarakis S., Dekkers O.M. (2016). Management of Adrenal Incidentalomas: European Society of Endocrinology Clinical Practice Guideline in Collaboration with the European Network for the Study of Adrenal Tumors. Eur. J. Endocrinol..

[27] Agarwal S.K. (2013). Multiple Endocrine Neoplasia Type 1. Front. Horm. Res..

[28] Gatta-Cherifi B., Chabre O., Murat A., Niccoli P., Cardot-Bauters C., Rohmer V., Young J., Delemer B., Du Boullay H., Verger M.F. (2012). Adrenal Involvement in MEN1. Analysis of 715 Cases from the Groupe d’etude Des Tumeurs Endocrines Database. Eur. J. Endocrinol. Eur. Fed. Endocr. Soc..

[29] Langer P., Cupisti K., Bartsch D.K., Nies C., Goretzki P.E., Rothmund M., Röher H.D. (2002). Adrenal Involvement in Multiple Endocrine Neoplasia Type 1. World J. Surg..

[30] Waldmann J., Bartsch D.K., Kann P.H., Fendrich V., Rothmund M., Langer P. (2007). Adrenal Involvement in Multiple Endocrine Neoplasia Type 1: Results of 7 Years Prospective Screening. Langenbecks Arch. Surg. Dtsch. Ges. Für Chir..

[31] Yoshida M., Hiroi M., Imai T., Kikumori T., Himeno T., Nakamura Y., Sasano H., Yamada M., Murakami Y., Nakamura S. (2011). A Case of ACTH-Independent Macronodular Adrenal Hyperplasia Associated with Multiple Endocrine Neoplasia Type 1. Endocr. J..

[32] Harding B., Lemos M.C., Reed A.A.C., Walls G.V., Jeyabalan J., Bowl M.R., Tateossian H., Sullivan N., Hough T., Fraser W.D. (2009). Multiple Endocrine Neoplasia Type 1 Knockout Mice Develop Parathyroid, Pancreatic, Pituitary and Adrenal Tumours with Hypercalcaemia, Hypophosphataemia and Hypercorticosteronaemia. Endocr. Relat. Cancer.

[33] Matyakhina L., Freedman R.J., Bourdeau I., Wei M.-H., Stergiopoulos S.G., Chidakel A., Walther M., Abu-Asab M., Tsokos M., Keil M. (2005). Hereditary Leiomyomatosis Associated with Bilateral, Massive, Macronodular Adrenocortical Disease and Atypical Cushing Syndrome: A Clinical and Molecular Genetic Investigation. J. Clin. Endocrinol. Metab..

[34] Shuch B., Ricketts C.J., Vocke C.D., Valera V.A., Chen C.C., Gautam R., Gupta G.N., Gomez Macias G.S., Merino M.J., Bratslavsky G. (2013). Adrenal Nodular Hyperplasia in Hereditary Leiomyomatosis and Renal Cell Cancer. J. Urol..

[35] Hsiao H.-P., Kirschner L.S., Bourdeau I., Keil M.F., Boikos S.A., Verma S., Robinson-White A.J., Nesterova M., Lacroix A., Stratakis C.A. (2009). Clinical and Genetic Heterogeneity, Overlap with Other Tumor Syndromes, and Atypical Glucocorticoid Hormone Secretion in Adrenocorticotropin-Independent Macronodular Adrenal Hyperplasia Compared with Other Adrenocortical Tumors. J. Clin. Endocrinol. Metab..

[36] Half E., Bercovich D., Rozen P. (2009). Familial Adenomatous Polyposis. Orphanet J. Rare Dis..

[37] Gaujoux S., Pinson S., Gimenez-Roqueplo A.-P., Amar L., Ragazzon B., Launay P., Meatchi T., Libé R., Bertagna X., Audebourg A. (2010). Inactivation of the APC Gene Is Constant in Adrenocortical Tumors from Patients with Familial Adenomatous Polyposis but Not Frequent in Sporadic Adrenocortical Cancers. Clin. Cancer Res. Off. J. Am. Assoc. Cancer Res..

[38] Hosogi H., Nagayama S., Kanamoto N., Yoshizawa A., Suzuki T., Nakao K., Sakai Y. (2009). Biallelic APC Inactivation Was Responsible for Functional Adrenocortical Adenoma in Familial Adenomatous Polyposis with Novel Germline Mutation of the APC Gene: Report of a Case. Jpn. J. Clin. Oncol..

[39] Brioude F., Kalish J.M., Mussa A., Foster A.C., Bliek J., Ferrero G.B., Boonen S.E., Cole T., Baker R., Bertoletti M. (2018). Expert Consensus Document: Clinical and Molecular Diagnosis, Screening and Management of Beckwith-Wiedemann Syndrome: An International Consensus Statement. Nat. Rev. Endocrinol..

[40] Carney J.A., Ho J., Kitsuda K., Young W.F.J., Stratakis C.A. (2012). Massive Neonatal Adrenal Enlargement Due to Cytomegaly, Persistence of the Transient Cortex, and Hyperplasia of the Permanent Cortex: Findings in Cushing Syndrome Associated with Hemihypertrophy. Am. J. Surg. Pathol..

[41] Matyakhina L., Pack S., Kirschner L.S., Pak E., Mannan P., Jaikumar J., Taymans S.E., Sandrini F., Carney J.A., Stratakis C.A. (2003). Chromosome 2 (2p16) Abnormalities in Carney Complex Tumours. J. Med. Genet..

[42] Kirschner L.S., Carney J.A., Pack S.D., Taymans S.E., Giatzakis C., Cho Y.S., Cho-Chung Y.S., Stratakis C.A. (2000). Mutations of the Gene Encoding the Protein Kinase A Type I-Alpha Regulatory Subunit in Patients with the Carney Complex. Nat. Genet..

[43] Casey M., Vaughan C.J., He J., Hatcher C.J., Winter J.M., Weremowicz S., Montgomery K., Kucherlapati R., Morton C.C., Basson C.T. (2000). Mutations in the Protein Kinase A R1alpha Regulatory Subunit Cause Familial Cardiac Myxomas and Carney Complex. J. Clin. Investig..

[44] Horvath A., Bertherat J., Groussin L., Guillaud-Bataille M., Tsang K., Cazabat L., Libé R., Remmers E., René-Corail F., Faucz F.R. (2010). Mutations and Polymorphisms in the Gene Encoding Regulatory Subunit Type 1-Alpha of Protein Kinase A (PRKAR1A): An Update. Hum. Mutat..

[45] Horvath A., Bossis I., Giatzakis C., Levine E., Weinberg F., Meoli E., Robinson-White A., Siegel J., Soni P., Groussin L. (2008). Large Deletions of the PRKAR1A Gene in Carney Complex. Clin. Cancer Res. Off. J. Am. Assoc. Cancer Res..

[46] Groussin L., Kirschner L.S., Vincent-Dejean C., Perlemoine K., Jullian E., Delemer B., Zacharieva S., Pignatelli D., Carney J.A., Luton J.P. (2002). Molecular Analysis of the Cyclic AMP-Dependent Protein Kinase A (PKA) Regulatory Subunit 1A (PRKAR1A) Gene in Patients with Carney Complex and Primary Pigmented Nodular Adrenocortical Disease (PPNAD) Reveals Novel Mutations and Clues for Pathophysiology: Augmented PKA Signaling Is Associated with Adrenal Tumorigenesis in PPNAD. Am. J. Hum. Genet..

[47] Patronas Y., Horvath A., Greene E., Tsang K., Bimpaki E., Haran M., Nesterova M., Stratakis C.A. (2012). In Vitro Studies of Novel PRKAR1A Mutants That Extend the Predicted RIα Protein Sequence into the 3′-Untranslated Open Reading Frame: Proteasomal Degradation Leads to RIα Haploinsufficiency and Carney Complex. J. Clin. Endocrinol. Metab..

[48] Amieux P.S., Howe D.G., Knickerbocker H., Lee D.C., Su T., Laszlo G.S., Idzerda R.L., McKnight G.S. (2002). Increased Basal CAMP-Dependent Protein Kinase Activity Inhibits the Formation of Mesoderm-Derived Structures in the Developing Mouse Embryo. J. Biol. Chem..

[49] Kirschner L.S., Kusewitt D.F., Matyakhina L., Towns W.H., Carney J.A., Westphal H., Stratakis C.A. (2005). A Mouse Model for the Carney Complex Tumor Syndrome Develops Neoplasia in Cyclic AMP-Responsive Tissues. Cancer Res..

[50] Griffin K.J., Kirschner L.S., Matyakhina L., Stergiopoulos S., Robinson-White A., Lenherr S., Weinberg F.D., Claflin E., Meoli E., Cho-Chung Y.S. (2004). Down-Regulation of Regulatory Subunit Type 1A of Protein Kinase A Leads to Endocrine and Other Tumors. Cancer Res..

[51] Sahut-Barnola I., de Joussineau C., Val P., Lambert-Langlais S., Damon C., Lefrançois-Martinez A.-M., Pointud J.-C., Marceau G., Sapin V., Tissier F. (2010). Cushing’s Syndrome and Fetal Features Resurgence in Adrenal Cortex-Specific Prkar1a Knockout Mice. PLoS Genet..

[52] Dumontet T., Sahut-Barnola I., Septier A., Montanier N., Plotton I., Roucher-Boulez F., Ducros V., Lefrançois-Martinez A.-M., Pointud J.-C., Zubair M. (2018). PKA Signaling Drives Reticularis Differentiation and Sexually Dimorphic Adrenal Cortex Renewal. JCI Insight.

[53] Mavrakis M., Lippincott-Schwartz J., Stratakis C.A., Bossis I. (2006). Depletion of Type IA Regulatory Subunit (RIalpha) of Protein Kinase A (PKA) in Mammalian Cells and Tissues Activates MTOR and Causes Autophagic Deficiency. Hum. Mol. Genet..

[54] Beuschlein F., Fassnacht M., Assié G., Calebiro D., Stratakis C.A., Osswald A., Ronchi C.L., Wieland T., Sbiera S., Faucz F.R. (2014). Constitutive Activation of PKA Catalytic Subunit in Adrenal Cushing’s Syndrome. N. Engl. J. Med..

[55] Lodish M.B., Yuan B., Levy I., Braunstein G.D., Lyssikatos C., Salpea P., Szarek E., Karageorgiadis A.S., Belyavskaya E., Raygada M. (2015). Germline PRKACA Amplification Causes Variable Phenotypes That May Depend on the Extent of the Genomic Defect: Molecular Mechanisms and Clinical Presentations. Eur. J. Endocrinol. Eur. Fed. Endocr. Soc..

[56] Swords F.M., Baig A., Malchoff D.M., Malchoff C.D., Thorner M.O., King P.J., Hunyady L., Clark A.J.L. (2002). Impaired Desensitization of a Mutant Adrenocorticotropin Receptor Associated with Apparent Constitutive Activity. Mol. Endocrinol. Baltim. Md.

[57] Swords F.M., Noon L.A., King P.J., Clark A.J.L. (2004). Constitutive Activation of the Human ACTH Receptor Resulting from a Synergistic Interaction between Two Naturally Occurring Missense Mutations in the MC2R Gene. Mol. Cell. Endocrinol..

[58] Libé R., Fratticci A., Coste J., Tissier F., Horvath A., Ragazzon B., Rene-Corail F., Groussin L., Bertagna X., Raffin-Sanson M.L. (2008). Phosphodiesterase 11A (PDE11A) and Genetic Predisposition to Adrenocortical Tumors. Clin. Cancer Res. Off. J. Am. Assoc. Cancer Res..

[59] Vezzosi D., Libé R., Baudry C., Rizk-Rabin M., Horvath A., Levy I., René-Corail F., Ragazzon B., Stratakis C.A., Vandecasteele G. (2012). Phosphodiesterase 11A (PDE11A) Gene Defects in Patients with Acth-Independent Macronodular Adrenal Hyperplasia (AIMAH): Functional Variants May Contribute to Genetic Susceptibility of Bilateral Adrenal Tumors. J. Clin. Endocrinol. Metab..

[60] Horvath A., Boikos S., Giatzakis C., Robinson-White A., Groussin L., Griffin K.J., Stein E., Levine E., Delimpasi G., Hsiao H.P. (2006). A Genome-Wide Scan Identifies Mutations in the Gene Encoding Phosphodiesterase 11A4 (PDE11A) in Individuals with Adrenocortical Hyperplasia. Nat. Genet..

[61] Horvath A., Mericq V., Stratakis C.A. (2008). Mutation in PDE8B, a Cyclic AMP-Specific Phosphodiesterase in Adrenal Hyperplasia. N. Engl. J. Med..

[62] Libé R., Horvath A., Vezzosi D., Fratticci A., Coste J., Perlemoine K., Ragazzon B., Guillaud-Bataille M., Groussin L., Clauser E. (2011). Frequent Phosphodiesterase 11A Gene (PDE11A) Defects in Patients with Carney Complex (CNC) Caused by PRKAR1A Mutations: PDE11A May Contribute to Adrenal and Testicular Tumors in CNC as a Modifier of the Phenotype. J. Clin. Endocrinol. Metab..

[63] Weinstein L.S., Shenker A., Gejman P.V., Merino M.J., Friedman E., Spiegel A.M. (1991). Activating Mutations of the Stimulatory G Protein in the McCune-Albright Syndrome. N. Engl. J. Med..

[64] Carney J.A., Young W.F., Stratakis C.A. (2011). Primary Bimorphic Adrenocortical Disease: Cause of Hypercortisolism in McCune-Albright Syndrome. Am. J. Surg. Pathol..

[65] Almeida M.Q., Azevedo M.F., Xekouki P., Bimpaki E.I., Horvath A., Collins M.T., Karaviti L.P., Jeha G.S., Bhattacharyya N., Cheadle C. (2012). Activation of Cyclic AMP Signaling Leads to Different Pathway Alterations in Lesions of the Adrenal Cortex Caused by Germline PRKAR1A Defects versus Those Due to Somatic GNAS Mutations. J. Clin. Endocrinol. Metab..

[66] Lacroix A., Bolté E., Tremblay J., Dupré J., Poitras P., Fournier H., Garon J., Garrel D., Bayard F., Taillefer R. (1992). Gastric Inhibitory Polypeptide-Dependent Cortisol Hypersecretion--a New Cause of Cushing’s Syndrome. N. Engl. J. Med..

[67] Reznik Y., Allali-Zerah V., Chayvialle J.A., Leroyer R., Leymarie P., Travert G., Lebrethon M.C., Budi I., Balliere A.M., Mahoudeau J. (1992). Food-Dependent Cushing’s Syndrome Mediated by Aberrant Adrenal Sensitivity to Gastric Inhibitory Polypeptide. N. Engl. J. Med..

[68] Lacroix A., Bourdeau I., Lampron A., Mazzuco T.L., Tremblay J., Hamet P. (2010). Aberrant G-Protein Coupled Receptor Expression in Relation to Adrenocortical Overfunction. Clin. Endocrinol. (Oxf.).

[69] Lacroix A., Ndiaye N., Tremblay J., Hamet P. (2001). Ectopic and Abnormal Hormone Receptors in Adrenal Cushing’s Syndrome. Endocr. Rev..

[70] Libé R., Coste J., Guignat L., Tissier F., Lefebvre H., Barrande G., Ajzenberg C., Tauveron I., Clauser E., Dousset B. (2010). Aberrant Cortisol Regulations in Bilateral Macronodular Adrenal Hyperplasia: A Frequent Finding in a Prospective Study of 32 Patients with Overt or Subclinical Cushing’s Syndrome. Eur. J. Endocrinol. Eur. Fed. Endocr. Soc..

[71] El Ghorayeb N., Bourdeau I., Lacroix A. (2015). Multiple Aberrant Hormone Receptors in Cushing’s Syndrome. Eur. J. Endocrinol. Eur. Fed. Endocr. Soc..

[72] Groussin L., Perlemoine K., Contesse V., Lefebvre H., Tabarin A., Thieblot P., Schlienger J.L., Luton J.P., Bertagna X., Bertherat J. (2002). The Ectopic Expression of the Gastric Inhibitory Polypeptide Receptor Is Frequent in Adrenocorticotropin-Independent Bilateral Macronodular Adrenal Hyperplasia, but Rare in Unilateral Tumors. J. Clin. Endocrinol. Metab..

[73] Assie G., Louiset E., Sturm N., René-Corail F., Groussin L., Bertherat J., Thomas M., Lefebvre H., Feige J.-J., Clauser E. (2010). Systematic Analysis of G Protein-Coupled Receptor Gene Expression in Adrenocorticotropin-Independent Macronodular Adrenocortical Hyperplasia Identifies Novel Targets for Pharmacological Control of Adrenal Cushing’s Syndrome. J. Clin. Endocrinol. Metab..

[74] Lampron A., Bourdeau I., Hamet P., Tremblay J., Lacroix A. (2006). Whole Genome Expression Profiling of Glucose-Dependent Insulinotropic Peptide (GIP)- and Adrenocorticotropin-Dependent Adrenal Hyperplasias Reveals Novel Targets for the Study of GIP-Dependent Cushing’s Syndrome. J. Clin. Endocrinol. Metab..

[75] Bertherat J., Contesse V., Louiset E., Barrande G., Duparc C., Groussin L., Emy P., Bertagna X., Kuhn J.-M., Vaudry H. (2005). In Vivo and in Vitro Screening for Illegitimate Receptors in Adrenocorticotropin-Independent Macronodular Adrenal Hyperplasia Causing Cushing’s Syndrome: Identification of Two Cases of Gonadotropin/Gastric Inhibitory Polypeptide-Dependent Hypercortisolism. J. Clin. Endocrinol. Metab..

[76] Louiset E., Contesse V., Groussin L., Cartier D., Duparc C., Barrande G., Bertherat J., Vaudry H., Lefebvre H. (2006). Expression of Serotonin7 Receptor and Coupling of Ectopic Receptors to Protein Kinase A and Ionic Currents in Adrenocorticotropin-Independent Macronodular Adrenal Hyperplasia Causing Cushing’s Syndrome. J. Clin. Endocrinol. Metab..

[77] Hofland J., Hofland L.J., van Koetsveld P.M., Steenbergen J., de Herder W.W., van Eijck C.H., de Krijger R.R., van Nederveen F.H., van Aken M.O., de Groot J.W. (2013). ACTH-Independent Macronodular Adrenocortical Hyperplasia Reveals Prevalent Aberrant in Vivo and in Vitro Responses to Hormonal Stimuli and Coupling of Arginine-Vasopressin Type 1a Receptor to 11β-Hydroxylase. Orphanet J. Rare Dis..

[78] Mannelli M., Ferruzzi P., Luciani P., Crescioli C., Buci L., Corona G., Serio M., Peri A. (2003). Cushing’s Syndrome in a Patient with Bilateral Macronodular Adrenal Hyperplasia Responding to Cisapride: An in Vivo and in Vitro Study. J. Clin. Endocrinol. Metab..

[79] Mazzuco T.L., Chabre O., Feige J.-J., Thomas M. (2006). Aberrant Expression of Human Luteinizing Hormone Receptor by Adrenocortical Cells Is Sufficient to Provoke Both Hyperplasia and Cushing’s Syndrome Features. J. Clin. Endocrinol. Metab..

[80] Mazzuco T.L., Chabre O., Sturm N., Feige J.-J., Thomas M. (2006). Ectopic Expression of the Gastric Inhibitory Polypeptide Receptor Gene Is a Sufficient Genetic Event to Induce Benign Adrenocortical Tumor in a Xenotransplantation Model. Endocrinology.

[81] Dall’Asta C., Ballarè E., Mantovani G., Ambrosi B., Spada A., Barbetta L., Colombo P., Travaglini P., Loli P., Beck-Peccoz P. (2004). Assessing the Presence of Abnormal Regulation of Cortisol Secretion by Membrane Hormone Receptors: In Vivo and in Vitro Studies in Patients with Functioning and Non-Functioning Adrenal Adenoma. Horm. Metab. Res. Horm. Stoffwechselforschung Horm. Métabolisme.

[82] Berthon A., Faucz F.R., Espiard S., Drougat L., Bertherat J., Stratakis C.A. (2017). Age-Dependent Effects of Armc5 Haploinsufficiency on Adrenocortical Function. Hum. Mol. Genet..

[83] Lecoq A.-L., Stratakis C.A., Viengchareun S., Chaligné R., Tosca L., Deméocq V., Hage M., Berthon A., Faucz F.R., Hanna P. (2017). Adrenal GIPR Expression and Chromosome 19q13 Microduplications in GIP-Dependent Cushing’s Syndrome. JCI Insight.

[84] Vaczlavik A., Bouys L., Letouze E., Perlemoine K., Guignat L., Letourneur F., Sibony M., Bonnet F., Heurtier V., Espiard S. (2020). Integrated Genomics Reveals Different Subgroups of Primary Bilateral Macronodular Adrenal Hyperplasia (PBMAH). Endocrine Abstracts.

[85] Assié G., Libé R., Espiard S., Rizk-Rabin M., Guimier A., Luscap W., Barreau O., Lefèvre L., Sibony M., Guignat L. (2013). ARMC5 Mutations in Macronodular Adrenal Hyperplasia with Cushing’s Syndrome. N. Engl. J. Med..

[86] Faucz F.R., Zilbermint M., Lodish M.B., Szarek E., Trivellin G., Sinaii N., Berthon A., Libé R., Assié G., Espiard S. (2014). Macronodular Adrenal Hyperplasia Due to Mutations in an Armadillo Repeat Containing 5 (ARMC5) Gene: A Clinical and Genetic Investigation. J. Clin. Endocrinol. Metab..

[87] Albiger N.M., Regazzo D., Rubin B., Ferrara A.M., Rizzati S., Taschin E., Ceccato F., Arnaldi G., Pecori Giraldi F., Stigliano A. (2016). A Multicenter Experience on the Prevalence of ARMC5 Mutations in Patients with Primary Bilateral Macronodular Adrenal Hyperplasia: From Genetic Characterization to Clinical Phenotype. Endocrine.

[88] Alencar G.A., Lerario A.M., Nishi M.Y., Mariani B.M.d.P., Almeida M.Q., Tremblay J., Hamet P., Bourdeau I., Zerbini M.C.N., Pereira M.A.A. (2014). ARMC5 Mutations Are a Frequent Cause of Primary Macronodular Adrenal Hyperplasia. J. Clin. Endocrinol. Metab..

[89] Kyo C., Usui T., Kosugi R., Torii M., Yonemoto T., Ogawa T., Kotani M., Tamura N., Yamamoto Y., Katabami T. (2019). ARMC5 Alterations in Primary Macronodular Adrenal Hyperplasia (PMAH) and the Clinical State of Variant Carriers. J. Endocr. Soc..

[90] Zilbermint M., Xekouki P., Faucz F.R., Berthon A., Gkourogianni A., Helene Schernthaner-Reiter M., Batsis M., Sinaii N., Quezado M.M., Merino M. (2015). Primary Aldosteronism and ARMC5 Variants. J. Clin. Endocrinol. Metab..

[91] Joseph J.J., Zhou X., Zilbermint M., Stratakis C.A., Faucz F.R., Lodish M.B., Berthon A., Wilson J.G., Hsueh W.A., Golden S.H. (2020). The Association of ARMC5 with the Renin-Angiotensin-Aldosterone System, Blood Pressure, and Glycemia in African Americans. J. Clin. Endocrinol. Metab..

[92] Bourdeau I., Oble S., Magne F., Lévesque I., Cáceres-Gorriti K.Y., Nolet S., Awadalla P., Tremblay J., Hamet P., Fragoso M.C.B.V. (2016). ARMC5 Mutations in a Large French-Canadian Family with Cortisol-Secreting β-Adrenergic/Vasopressin Responsive Bilateral Macronodular Adrenal Hyperplasia. Eur. J. Endocrinol..

[93] Gagliardi L., Schreiber A.W., Hahn C.N., Feng J., Cranston T., Boon H., Hotu C., Oftedal B.E., Cutfield R., Adelson D.L. (2014). ARMC5 Mutations Are Common in Familial Bilateral Macronodular Adrenal Hyperplasia. J. Clin. Endocrinol. Metab..

[94] Elbelt U., Trovato A., Kloth M., Gentz E., Finke R., Spranger J., Galas D., Weber S., Wolf C., König K. (2014). Molecular and Clinical Evidence for an ARMC5 Tumor Syndrome: Concurrent Inactivating Germline and Somatic Mutations Are Associated with Both Primary Macronodular Adrenal Hyperplasia and Meningioma. J. Clin. Endocrinol. Metab..

[95] Jojima T., Kogai T., Iijima T., Kato K., Sagara M., Kezuka A., Kase M., Sakurai S., Akimoto K., Sakumoto J. (2020). Genetic Alteration of ARMC5 in a Patient Diagnosed with Meningioma and Primary Macronodular Adrenal Hyperplasia: A Case Report. Eur. J. Endocrinol..

[96] Correa R., Zilbermint M., Berthon A., Espiard S., Batsis M., Papadakis G.Z., Xekouki P., Lodish M.B., Bertherat J., Faucz F.R. (2015). The ARMC5 Gene Shows Extensive Genetic Variance in Primary Macronodular Adrenocortical Hyperplasia. Eur. J. Endocrinol..

[97] Drougat L., Espiard S., Bertherat J. (2015). Genetics of Primary Bilateral Macronodular Adrenal Hyperplasia: A Model for Early Diagnosis of Cushing’s Syndrome?. Eur. J. Endocrinol. Eur. Fed. Endocr. Soc..

[98] Suzuki S., Tatsuno I., Oohara E., Nakayama A., Komai E., Shiga A., Kono T., Takiguchi T., Higuchi S., Sakuma I. (2015). GERMLINE DELETION OF ARMC5 IN FAMILIAL PRIMARY MACRONODULAR ADRENAL HYPERPLASIA. Endocr. Pract. Off. J. Am. Coll. Endocrinol. Am. Assoc. Clin. Endocrinol..

[99] Berthon A., Faucz F., Bertherat J., Stratakis C.A. (2017). Analysis of ARMC5 Expression in Human Tissues. Mol. Cell. Endocrinol..

[100] Cavalcante I.P., Nishi M., Zerbini M.C.N., Almeida M.Q., Brondani V.B., Botelho M.L.A.d.A., Tanno F.Y., Srougi V., Chambo J.L., Mendonca B.B. (2018). The Role of ARMC5 in Human Cell Cultures from Nodules of Primary Macronodular Adrenocortical Hyperplasia (PMAH). Mol. Cell. Endocrinol..

[101] Bourdeau I., Antonini S.R., Lacroix A., Kirschner L.S., Matyakhina L., Lorang D., Libutti S.K., Stratakis C.A. (2004). Gene Array Analysis of Macronodular Adrenal Hyperplasia Confirms Clinical Heterogeneity and Identifies Several Candidate Genes as Molecular Mediators. Oncogene.

[102] Wurth R., Tirosh A., Kamilaris C.D.C., Camacho J., Faucz F.R., Maria A.G., Berthon A., Papadakis G.Z., Nilubol N., Hamimi A. (2021). Volumetric Modeling of Adrenal Gland Size in Primary Bilateral Macronodular Adrenocortical Hyperplasia. J. Endocr. Soc..

[103] Hu Y., Lao L., Mao J., Jin W., Luo H., Charpentier T., Qi S., Peng J., Hu B., Marcinkiewicz M.M. (2017). Armc5 Deletion Causes Developmental Defects and Compromises T-Cell Immune Responses. Nat. Commun..

[104] Almeida M.Q., Harran M., Bimpaki E.I., Hsiao H.-P., Horvath A., Cheadle C., Watkins T., Nesterova M., Stratakis C.A. (2011). Integrated Genomic Analysis of Nodular Tissue in Macronodular Adrenocortical Hyperplasia: Progression of Tumorigenesis in a Disorder Associated with Multiple Benign Lesions. J. Clin. Endocrinol. Metab..

[105] Cavalcante I.P., Vaczlavik A., Drougat L., Lotfi C.F.P., Perlemoine K., Ribes C., Rizk-Rabin M., Clauser E., Fragoso M.C.B.V., Bertherat J. (2020). Cullin 3 Targets the Tumor Suppressor Gene ARMC5 for Ubiquitination and Degradation. Endocr. Relat. Cancer.

[106] Ehrhart-Bornstein M., Hinson J.P., Bornstein S.R., Scherbaum W.A., Vinson G.P. (1998). Intraadrenal Interactions in the Regulation of Adrenocortical Steroidogenesis. Endocr. Rev..

[107] Perraudin V., Delarue C., Lefebvre H., Contesse V., Kuhn J.M., Vaudry H. (1993). Vasopressin Stimulates Cortisol Secretion from Human Adrenocortical Tissue through Activation of V1 Receptors. J. Clin. Endocrinol. Metab..

[108] Lefebvre H., Duparc C., Prévost G., Bertherat J., Louiset E. (2015). Cell-to-Cell Communication in Bilateral Macronodular Adrenal Hyperplasia Causing Hypercortisolism. Front. Endocrinol..

[109] Suda T., Tomori N., Tozawa F., Demura H., Shizume K., Mouri T., Miura Y., Sasano N. (1984). Immunoreactive Corticotropin and Corticotropin-Releasing Factor in Human Hypothalamus, Adrenal, Lung Cancer, and Pheochromocytoma. J. Clin. Endocrinol. Metab..

[110] Louiset E., Duparc C., Young J., Renouf S., Tetsi Nomigni M., Boutelet I., Libé R., Bram Z., Groussin L., Caron P. (2013). Intraadrenal Corticotropin in Bilateral Macronodular Adrenal Hyperplasia. N. Engl. J. Med..

[111] Lacroix A. (2013). Heredity and Cortisol Regulation in Bilateral Macronodular Adrenal Hyperplasia. N. Engl. J. Med..

[112] Lefebvre H., Contesse V., Delarue C., Feuilloley M., Hery F., Grise P., Raynaud G., Verhofstad A.A., Wolf L.M., Vaudry H. (1992). Serotonin-Induced Stimulation of Cortisol Secretion from Human Adrenocortical Tissue Is Mediated through Activation of a Serotonin4 Receptor Subtype. Neuroscience.

[113] Nieman L.K., Biller B.M.K., Findling J.W., Murad M.H., Newell-Price J., Savage M.O., Tabarin A. (2015). Endocrine Society Treatment of Cushing’s Syndrome: An Endocrine Society Clinical Practice Guideline. J. Clin. Endocrinol. Metab..

[114] Ngaosuwan K., Johnston D.G., Godsland I.F., Cox J., Majeed A., Quint J.K., Oliver N., Robinson S. (2021). Cardiovascular Disease in Patients With Primary and Secondary Adrenal Insufficiency and the Role of Comorbidities. J. Clin. Endocrinol. Metab..

[115] Tresoldi A.S., Sumilo D., Perrins M., Toulis K.A., Prete A., Reddy N., Wass J.A.H., Arlt W., Nirantharakumar K. (2020). Increased Infection Risk in Addison’s Disease and Congenital Adrenal Hyperplasia. J. Clin. Endocrinol. Metab..

[116] Johannsson G., Falorni A., Skrtic S., Lennernäs H., Quinkler M., Monson J.P., Stewart P.M. (2015). Adrenal Insufficiency: Review of Clinical Outcomes with Current Glucocorticoid Replacement Therapy. Clin. Endocrinol. (Oxf.).

[117] Nieman L.K. (2015). Update on Subclinical Cushing’s Syndrome. Curr. Opin. Endocrinol. Diabetes Obes..

[118] Bancos I., Alahdab F., Crowley R.K., Chortis V., Delivanis D.A., Erickson D., Natt N., Terzolo M., Arlt W., Young W.F.J. (2016). THERAPY OF ENDOCRINE DISEASE: Improvement of Cardiovascular Risk Factors after Adrenalectomy in Patients with Adrenal Tumors and Subclinical Cushing’s Syndrome: A Systematic Review and Meta-Analysis. Eur. J. Endocrinol..

[119] Powell A.C., Stratakis C.A., Patronas N.J., Steinberg S.M., Batista D., Alexander H.R., Pingpank J.F., Keil M., Bartlett D.L., Libutti S.K. (2008). Operative Management of Cushing Syndrome Secondary to Micronodular Adrenal Hyperplasia. Surgery.

[120] Meloche-Dumas L., Mercier F., Lacroix A. (2021). Role of Unilateral Adrenalectomy in Bilateral Adrenal Hyperplasias with Cushing’s Syndrome. Best Pract. Res. Clin. Endocrinol. Metab..

[121] Zhang Y., Li H. (2015). Classification and Surgical Treatment for 180 Cases of Adrenocortical Hyperplastic Disease. Int. J. Clin. Exp. Med..

[122] Carney J.A., Young W.F. (1992). Primary Pigmented Nodular Adrenocortical Disease and Its Associated Conditions. The Endocrinologist.

[123] Tadjine M., Lampron A., Ouadi L., Horvath A., Stratakis C.A., Bourdeau I. (2008). Detection of Somatic Beta-Catenin Mutations in Primary Pigmented Nodular Adrenocortical Disease (PPNAD). Clin. Endocrinol. (Oxf.).

[124] Sarlis N.J., Chrousos G.P., Doppman J.L., Carney J.A., Stratakis C.A. (1997). Primary Pigmented Nodular Adrenocortical Disease: Reevaluation of a Patient with Carney Complex 27 Years after Unilateral Adrenalectomy. J. Clin. Endocrinol. Metab..

[125] Cohen M.M. (2006). The New Bone Biology: Pathologic, Molecular, and Clinical Correlates. Am. J. Med. Genet. A.

[126] Guanà R., Gesmundo R., Morino M., Matarazzo P., Pucci A., Pasini B., Lala R., Fiore L., Repici M., Canavese F. (2010). Laparoscopic Unilateral Adrenalectomy in Children for Isolated Primary Pigmented Nodular Adrenocortical Disease (PPNAD): Case Report and Literature Review. Eur. J. Pediatr. Surg. Off. J. Austrian Assoc. Pediatr. Surg. Al Z. Kinderchir..

[127] Xu Y., Rui W., Qi Y., Zhang C., Zhao J., Wang X., Wu Y., Zhu Q., Shen Z., Ning G. (2013). The Role of Unilateral Adrenalectomy in Corticotropin-Independent Bilateral Adrenocortical Hyperplasias. World J. Surg..

[128] Kyrilli A., Lytrivi M., Bouquegneau M.S., Demetter P., Lucidi V., Garcia C., Moreno-Reyes R., Tabarin A., Corvilain B., Driessens N. (2019). Unilateral Adrenalectomy Could Be a Valid Option for Primary Nodular Adrenal Disease: Evidence From Twins. J. Endocr. Soc..

[129] Kumorowicz-Czoch M., Dolezal-Oltarzewska K., Roztoczynska D., Chrupek M., Prokurat A.I., Drabik G., Starzyk J. (2011). Causes and Consequences of Abandoning One-Stage Bilateral Adrenalectomy Recommended in Primary Pigmented Nodular Adrenocortical Disease--Case Presentation. J. Pediatr. Endocrinol. Metab. JPEM.

[130] Debillon E., Velayoudom-Cephise F.-L., Salenave S., Caron P., Chaffanjon P., Wagner T., Massoutier M., Lambert B., Benoit M., Young J. (2015). Unilateral Adrenalectomy as a First-Line Treatment of Cushing’s Syndrome in Patients With Primary Bilateral Macronodular Adrenal Hyperplasia. J. Clin. Endocrinol. Metab..

[131] Acharya R., Dhir M., Bandi R., Yip L., Challinor S. (2019). Outcomes of Adrenal Venous Sampling in Patients with Bilateral Adrenal Masses and ACTH-Independent Cushing’s Syndrome. World J. Surg..

[132] Ueland G.Å., Methlie P., Jøssang D.E., Sagen J.V., Viste K., Thordarson H.B., Heie A., Grytaas M., Løvås K., Biermann M. (2018). Adrenal Venous Sampling for Assessment of Autonomous Cortisol Secretion. J. Clin. Endocrinol. Metab..

[133] Osswald A., Quinkler M., Di Dalmazi G., Deutschbein T., Rubinstein G., Ritzel K., Zopp S., Bertherat J., Beuschlein F., Reincke M. (2019). Long-Term Outcome of Primary Bilateral Macronodular Adrenocortical Hyperplasia After Unilateral Adrenalectomy. J. Clin. Endocrinol. Metab..

[134] Yoshiaki Tanno F., Srougi V., Almeida M.Q., Ide Yamauchi F., Morbeck Almeida Coelho F., Nishi M.Y., Claudia Nogueira Zerbini M., Silvia Correa Soares I., Adelaide Albergaria Pereira M., Laiz Silva Charchar H. (2020). A New Insight into the Surgical Treatment of Primary Macronodular Adrenal Hyperplasia. J. Endocr. Soc..

[135] Albiger N.M., Ceccato F., Zilio M., Barbot M., Occhi G., Rizzati S., Fassina A., Mantero F., Boscaro M., Iacobone M. (2015). An Analysis of Different Therapeutic Options in Patients with Cushing’s Syndrome Due to Bilateral Macronodular Adrenal Hyperplasia: A Single-Centre Experience. Clin. Endocrinol. (Oxf.).

[136] Mazzuco T.L., Chaffanjon P., Martinie M., Sturm N., Chabre O. (2009). Adrenal Cushing’s Syndrome Due to Bilateral Macronodular Adrenal Hyperplasia: Prediction of the Efficacy of Beta-Blockade Therapy and Interest of Unilateral Adrenalectomy. Endocr. J..

[137] Oki K., Yamane K., Nakanishi S., Nakashima R., Jitsuiki K., Kohno N. (2009). Improvement of Hypercortisolism by β-Blocker Therapy in Subclinical Cushing’s Syndrome Associated with ACTH-Independent Macronodular Adrenocortical Hyperplasia. Endocrine.

[138] Preumont V., Mermejo L.M., Damoiseaux P., Lacroix A., Maiter D. (2011). Transient Efficacy of Octreotide and Pasireotide (SOM230) Treatment in GIP-Dependent Cushing’s Syndrome. Horm. Metab. Res. Horm. Stoffwechselforschung Horm. Métabolisme.

[139] Karapanou O., Vlassopoulou B., Tzanela M., Stratigou T., Tsatlidis V., Tsirona S., Tsagarakis S. (2013). Adrenocorticotropic Hormone Independent Macronodular Adrenal Hyperplasia Due to Aberrant Receptor Expression: Is Medical Treatment Always an Option?. Endocr. Pract. Off. J. Am. Coll. Endocrinol. Am. Assoc. Clin. Endocrinol..

[140] Nagai M., Narita I., Omori K., Komura S., Arakawa M. (1999). Adrenocorticotropic Hormone-Independent Bilateral Adrenocortical Macronodular Hyperplasia Treated with Mitotane. Intern. Med. Tokyo Jpn..

[141] Comte-Perret S., Zanchi A., Gomez F. (2014). Long-Term Low-Dose Ketoconazole Treatment in Bilateral Macronodular Adrenal Hyperplasia. Endocrinol. Diabetes Metab. Case Rep..

[142] Campo M.R., Lamacchia O., Farese A., Conserva A., Picca G., Grilli G., Cignarelli M. (2015). Mitotane and Carney Complex: Ten Years Follow-up of a Low-Dose Mitotane Regimen Inducing a Sustained Correction of Hypercortisolism. Horm. Athens Greece.

[143] Debono M., Harrison R.F., Chadarevian R., Gueroult C., Abitbol J.-L., Newell-Price J. (2017). Resetting the Abnormal Circadian Cortisol Rhythm in Adrenal Incidentaloma Patients With Mild Autonomous Cortisol Secretion. J. Clin. Endocrinol. Metab..

